# Is the Ensemble Machine Learning Model a Reliable Method for Detecting Neoplastic Infiltration of Thyroid Cartilage in Laryngeal Cancers?

**DOI:** 10.3390/medicina61111945

**Published:** 2025-10-30

**Authors:** Sermin Can, Ömer Türk, Muhammed Ayral, Günay Kozan, Mehmet Önür, Eyyüp Yagız, Mehmet Akdag

**Affiliations:** 1 Department of Otorhinolaryngology and Head and Neck Surgery Clinic, Faculty of Medicine, Dicle University, 21010 Diyarbakır, Türkiye; drayral@hotmail.com (M.A.); gunaykozan@hotmail.com (G.K.); mehmetonurbatman@gmail.com (M.Ö.); mehmet.akdag@dicle.edu.tr (M.A.); 2 Department of Computer Engineering, Faculty of Engineering and Architecture, Mardin Artuklu University, 47100 Mardin, Türkiye; omerturk@artuklu.edu.tr; 3 Department of Otorhinolaryngology and Head and Neck Surgery Clinic, Sanlıurfa Training and Research Hospital, 63200 Sanlıurfa, Türkiye; yagizeyyup5621@gmail.com

**Keywords:** ensemble, invasion, larynx, machine learning, thyroid cartilage

## Abstract

*Background and Objectives*: We aimed to apply the ensemble machine learning model to diagnose thyroid cartilage invasion detected in computer tomography (CT) images in laryngeal cancers and evaluate the diagnostic performance of the model. *Materials and Methods*: A total of 313 patients were divided into two groups: the cartilage invasion group and the no cartilage invasion group. At least four CT slices were randomly selected for each patient, resulting in a total of 1251 images used in the study. A total of 619 axial CT images from the no cartilage invasion group and 632 axial CT images from the cartilage invasion group were used in the study. We reviewed the CT images and histopathological diagnoses in all cases to determine the invasion positive- or negative-status as a ground truth. The ensemble model, comprising ResNet50 and MobileNet deep learning architectures, was applied to CT images. *Results*: The following were obtained by the ensemble model with the test dataset: area under the curve (AUC) 0.99, and accuracy 96.54%. This model demonstrates a very high level of performance in detecting thyroid cartilage invasion. *Conclusions*: The ensemble machine learning model is an effective method for detecting neoplastic infiltration of the thyroid cartilage. Moreover, it may be a valuable diagnostic tool for clinicians in assessing disease prognosis and determining appropriate treatment strategies in laryngeal cancers. In conclusion, this model could be integrated into future clinical practice in laryngology and head and neck surgery for the detection of cartilage neoplastic infiltration.

## 1. Introduction

Laryngeal cancers are among the most prevalent malignancies in the head and neck region, associated with the highest mortality rates among these cancers. Approximately 85–95% of laryngeal cancers are histologically classified as squamous cell carcinoma. These malignancies are often diagnosed at advanced stages, significantly impacting prognosis and therapeutic outcomes [1]. Thyroid cartilage invasion plays a critical role in the primary tumor staging of laryngeal cancers [2]. According to the American Joint Committee on Cancer (AJCC) guidelines, focal invasion of the thyroid cartilage, involvement of the inner cortex, or invasion of the paraglottic or preepiglottic space without thyroid cartilage invasion is classified as T3 disease. In contrast, invasion of the outer cortex or evidence of extralaryngeal extension is categorized as T4 disease [3]. The detection of thyroid cartilage invasion plays a key role in determining the appropriate treatment strategy for advanced-stage laryngeal cancer. In T3-stage disease, characterized by focal cartilage invasion or invasion of the paraglottic or preepiglottic space without thyroid cartilage involvement, organ-preserving treatment approaches—such as partial laryngectomy or concomitant chemoradiotherapy—are generally preferred. In contrast, T4-stage disease, marked by extensive cartilage invasion, typically necessitates total laryngectomy. Additionally, the presence of neoplastic cartilage invasion may adversely affect the response to radiation therapy and is associated with a higher risk of tumor recurrence [4,5]. As a result, cartilage invasion in laryngeal carcinomas plays a significant role in both prognosis assessment and treatment selection. Therefore, accurate evaluation of cartilage invasion is essential for determining the most appropriate treatment for advanced-stage laryngeal cancers [6].

Both CT and Magnetic Resonance Imaging (MRI) are routinely used to assess cartilage invasion. However, during the interpretation of these imaging modalities, cartilage invasion may sometimes be overestimated, potentially leading to unnecessary total laryngectomies. Conversely, sometimes it may be underestimated or overlooked, resulting in suboptimal treatment decisions [7,8].

In recent years, artificial intelligence has been increasingly utilized in the field of radiology. It involves the use of highly complex methods to replicate certain cognitive functions based on computer technologies and algorithmic techniques [9]. Deep learning techniques such as convolutional neural networks have been employed for classification in imaging studies. Following evidence that their performance can match that of human experts, these techniques have garnered significant attention in recent times [10].

Given this information, in this study, we investigated whether an ensemble deep learning model integrating ResNet50 and MobileNet architectures could be used to detect thyroid cartilage invasion in CT images of laryngeal cancers. Unlike single-architecture models, ensemble approaches may leverage complementary feature extraction strengths to improve diagnostic accuracy. Through this approach, we aimed to apply the ensemble machine learning model to diagnose thyroid cartilage invasion detected in computer tomography (CT) images in laryngeal cancers and evaluate the diagnostic performance of the model.

## 2. Materials and Methods

The study was initiated following approval from the local ethics committee (14 May 2025, page 195). A total of 313 patients (288 males, 25 females; mean age, 62.5 ± 9.7 years; range, 41–84 years) who had been followed for laryngeal cancer between 2010 and 2022 were retrospectively reviewed in the study. At least four CT slices were randomly selected for each patient, resulting in a total of 1251 images used in the study. For each patient, at least four axial CT slices were selected according to predefined anatomical and image-quality criteria. The selected slices passed through the most representative portion of the thyroid cartilage and tumor, included the mid-cartilage level when available, and displayed at least one-fourth of the cartilage structure clearly within the image. Slices with motion artifacts, cystic changes, or poor image quality were excluded. This semi-standardized approach ensured consistent and clinically relevant image selection across all patients. The patients were divided into two groups: the cartilage invasion group and the no cartilage invasion group. A total of 155 patients who underwent partial laryngectomy and were found to have no histopathological evidence of thyroid cartilage invasion were included in the no cartilage invasion group. A total of 619 axial CT images from this group were used in the study. A total of 158 patients who underwent total laryngectomy and were histopathologically confirmed to have thyroid cartilage invasion were included in the cartilage invasion group. A total of 632 axial CT images from this group were used in the study. The mean age of patients in the cartilage invasion group was 63.4 ± 9.1 years, while that of the no cartilage invasion group was 61.7 ± 10.3 years, with no statistically significant difference between the two groups (*p* > 0.05). Most patients were male (92.0%) and had a positive history of smoking (88.8%). For the purposes of this study, patients with histopathologically confirmed thyroid cartilage invasion, regardless of the depth or extent of invasion, were collectively categorized into a single “cartilage invasion” group. This grouping included both T3-stage cases (focal or inner cortex invasion) and T4-stage cases (major or outer cortex invasion). The rationale for this approach was that the principal aim of the study was to assess the model’s performance in identifying the presence or absence of cartilage invasion on imaging, rather than in differentiating between the specific stages or degrees of invasion. As histopathological results provided a definitive reference standard for the existence of invasion, a binary classification framework (cartilage invasion vs. no cartilage invasion) was adopted to ensure methodological clarity and consistency. Inclusion criteria were as follows: (1) availability of comprehensive clinical data; (2) patients who had undergone total or partial laryngectomy, including both primary tumors and recurrences following radiotherapy or chemoradiotherapy; and (3) histopathological diagnosis of squamous cell carcinoma (SCC). Exclusion criteria were as follows: (1) absence of comprehensive clinical data; (2) presence of pathological findings other than SCC; and (3) CT images compromised by motion artifacts or beam-hardening artifacts caused by dental implants, as well as cases in which tumor lesions were not clearly identifiable on CT examinations.

### 2.1. CT Imaging Protocol

All CT images were obtained after an intravenous injection of contrast (100 mL iopamidol, Isovue 370; Bracco, Princeton, NJ, USA). These images were acquired from the axial and coronal planes using soft tissue and bone window settings (window width: 3000 Hounsfield Units, window level: 400–500 Hounsfield Units) extending from the skull base to the thoracic inlet and spaced 2 mm apart. At least four images were obtained from each patient to create more homogeneous data. Only one image was excluded due to poor quality.

### 2.2. Ensemble Model Recommended for Classification

**Figure 1:**
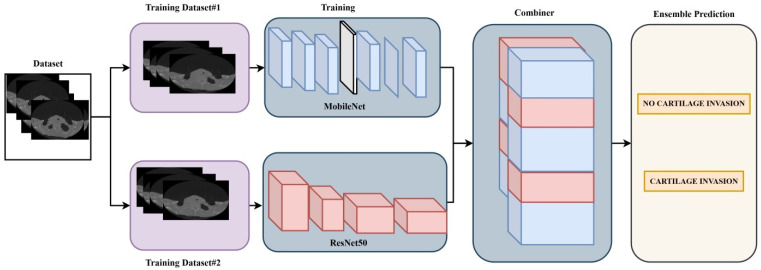
Ensemble model recommended for classification.

In the literature, various datasets have been classified using different deep learning architectures. One of the main objectives of such studies is to identify and develop the most accurate model applicable in this field. Unlike other architectures, ensemble models represent a technique that combines multiple classification models to improve overall performance [11]. In this study, ResNet50 and MobileNet deep learning architectures were used as an ensemble model. In this model, the pre-trained weights of the ResNet50 and MobileNet architectures were utilized through the transfer learning method [12,13,14]. A Dense layer with 1024 neurons was added to the final layer of each of the two deep learning architectures, and the classifier output was set to 2 due to the binary classification task. The learning rate for both deep learning architectures was set to 0.001. For each model, the batch size (number of images fed into the network per step) was set to 8, and the number of epochs was set to 10. The ensemble model implemented in the study is shown in Figure 1.

In the study, a total of 1251 images were used, including 619 from the no cartilage invasion class and 632 from the cartilage invasion class. Since these images were of varying sizes, they were resized to 224 × 224 pixels. Subsequently, the dataset was randomly split for training each deep learning model used in the ensemble model.

**Figure 2:**
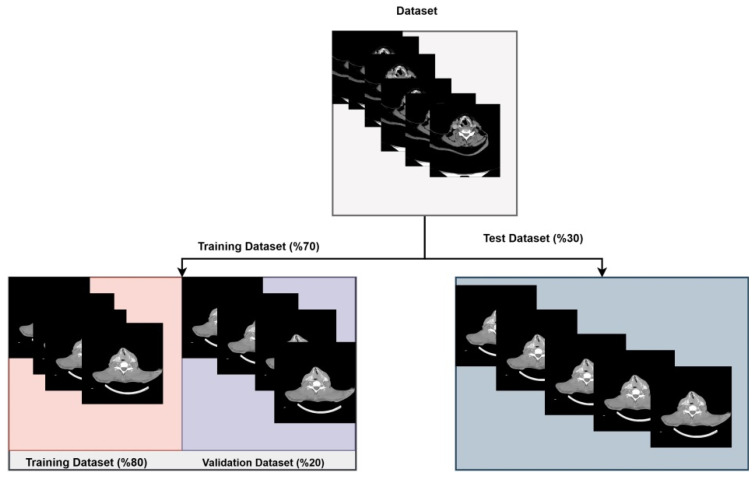
The distribution of the images used in the ensemble model.

Of the 1251 images used from the two classes, 70% were allocated to the training set and 30% to the test set. Furthermore, 20% of the images in the training set were used as validation data during the training process of the deep learning architectures. The distribution of the images used in the ensemble model is shown in Figure 2.

## 3. Results

Out of a total of 1251 patients, images from 875 patients were used for the training set, while images from 376 patients were used for the test set. After the training phase of the ensemble model was completed, testing was performed. The test accuracy of the model was found to be 96.54%. When evaluating deep learning models, accuracy is the primary metric to be considered, as it represents the ratio of correctly predicted cases to the total number of cases evaluated. Therefore, this metric is particularly important for classification models. In addition, the model’s precision, recall, and F1-score results were also obtained. Precision, recall, and F1-score statistical performance evaluation metrics were calculated to measure the performance of the study’s proposed models. The mathematical formulas for these metrics are as follows:

(1) $$Precision=\frac{TP}{TP+FP}$$

(2) $$Recall=\frac{TP}{TP+FN}$$

(3) $$F1\text{-}Score=2\times \frac{Precision\times Recall}{Precision+Recall}$$

The given metrics TP, TN, FP, and FN denote the true positive, true negative, false positive, and false negative of a confusion matrix, respectively, [14,15]. The precision and F1-scores of the ensemble model were obtained as 0.96, and the Recall metric was obtained as 0.97.

**Figure 3:**
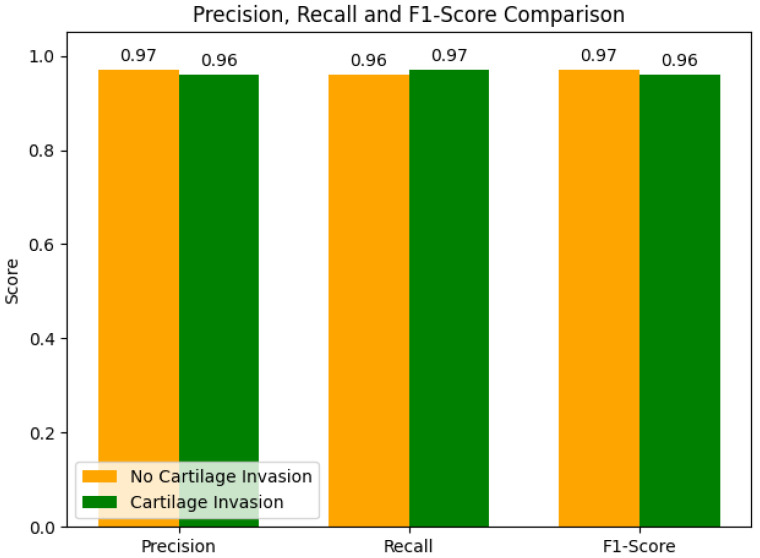
Performance metrics obtained from the Ensemble model for each group.

The precision, recall, and F1-score metrics obtained from the ensemble model for each group, along with their inter-group comparisons, are presented in Figure 3. In the comparison of performance metrics between the groups, for the no cartilage invasion group, the precision and F1-score performance metrics were 0.97, and the recall metric was 0.96. For the cartilage invasion group, the recall metric was 0.97, and the precision and F1-score performance metrics were 0.96.

**Figure 4:**
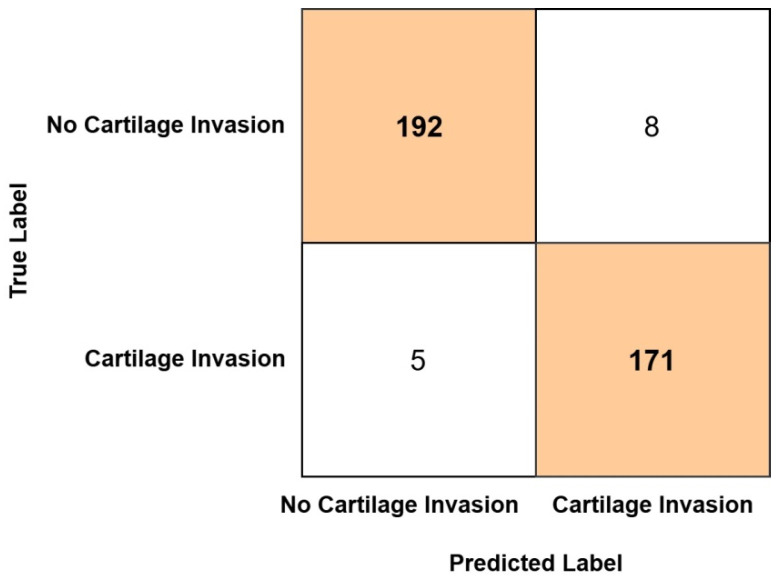
Confusion matrix obtained from the Ensemble model.

As a result of classification, the confusion matrix obtained from the Ensemble model is presented in Figure 4. As identified from the confusion matrix, among the 200 test set images belonging to the no cartilage invasion group, 192 were correctly classified, while 8 were misclassified as cartilage invasion. Similarly, of the 176 test set images in the cartilage invasion group, 171 were correctly classified, whereas 5 were incorrectly classified as no cartilage invasion.

**Figure 5:**
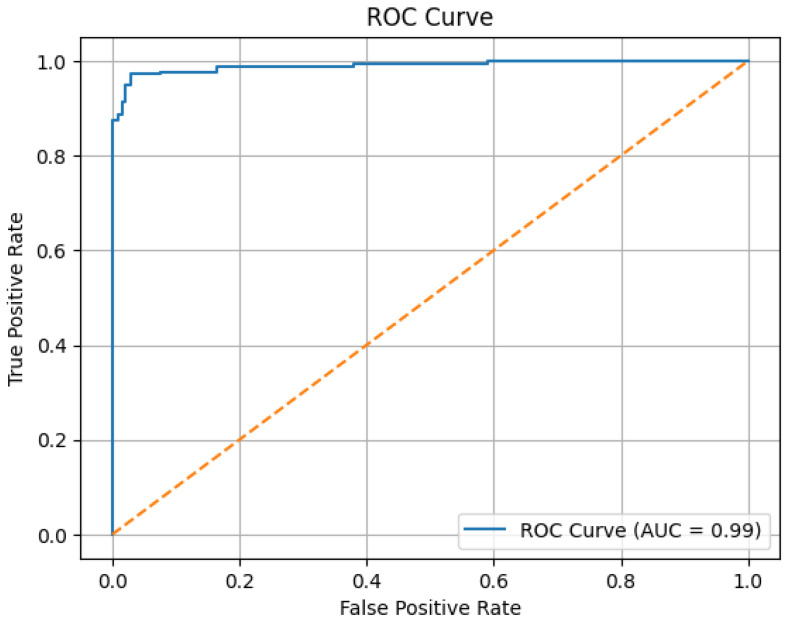
ROC curve obtained from the ensemble model.

The ROC (Receiver Operating Characteristic) curve obtained from the Ensemble model is shown in Figure 5. The ROC curve illustrates the true positive rates versus the false positive rates. The area under the curve (AUC) indicates the model’s ability to distinguish between the classes [16]. In this study, the proposed Ensemble model obtained an AUC value of 0.99, demonstrating its high effectiveness in distinguishing between the two classes.

## 4. Discussion

Despite advancements in technology, the mortality rate of laryngeal cancers continues to rise. These tumors are often diagnosed at an advanced locoregional stage [17,18]. Cartilage invasion plays a critical role in the staging and treatment selection of advanced-stage laryngeal carcinoma. Neoplastic infiltration of the laryngeal cartilages is the most common source of error in cancer staging assessment. It has been reported that neoplastic invasion of the cartilage may increase the risk of complications such as edema and necrosis following radiotherapy, as well as the likelihood of tumor recurrence. Consequently, it is associated with a negative impact on prognosis. Moreover, cartilage invasion is considered a contraindication not only for radiotherapy but also for partial surgical techniques [19]. Therefore, total laryngectomy is considered the most appropriate treatment option for these patients. In contrast, for patients without cartilage invasion, organ-preserving treatments (partial laryngectomy or chemoradiotherapy) are currently preferred as the optimal therapeutic approach. Due to these differences in treatment selection, accurate diagnosis of cartilage invasion requires a reliable diagnostic test [20]. Computed tomography (CT) and magnetic resonance imaging (MRI) are commonly used in the diagnosis of cartilage invasion. However, both imaging modalities have limitations, and their interpretation largely depends on the radiologist’s experience and expertise [21]. Additionally, some studies have reported that ultrasonographic examination can provide detailed anatomical reference points in cases of laryngeal tumor pathology [22]. Today, artificial intelligence (AI) is rapidly expanding in the field of healthcare, as it utilizes computer algorithms to perform tasks that typically require human intelligence [23]. Owing to the increasing availability of computer power, we aimed to use an ensemble model—a combined method derived from subsets of AI—in our study to assist in the diagnosis of thyroid cartilage invasion. Thus, we investigated whether this model could be used as a supportive diagnostic tool in advanced-stage laryngeal cancers, aiding both in treatment selection and in the determination of disease prognosis.

In our study, the ensemble model correctly classified 192 out of 200 patients without thyroid cartilage invasion and 171 out of 176 patients with thyroid cartilage invasion in the test set. Consequently, the model achieved an accuracy of 96.54% and an AUC value of 0.99 in the test set. These ratios indicate that the ensemble model exhibits a high level of diagnostic accuracy in detecting thyroid cartilage invasion in patients with laryngeal cancer. Based on these results, the findings support the potential clinical utility of the model as a reliable tool for assisting in the assessment of cartilage invasion.

Guo et al. developed artificial intelligence (AI) models based on CT-derived radiomic features (including the LR-SVMSMOTE model and the LR model) to detect thyroid cartilage invasion in laryngeal and hypopharyngeal carcinomas. In a cohort of 265 patients, they evaluated the predictive performance of both the AI models and radiologist assessments for detecting thyroid cartilage invasion. AUC values were reported as 0.905 for the AI model and 0.721 for the radiologist evaluations, respectively [24]. Similarly, Takano et al. applied a ResNet101 model to CT images of 91 patients with laryngeal and hypopharyngeal carcinoma. The model achieved an AUC of 0.82 and an accuracy of 90% in detecting thyroid cartilage invasion. Based on these findings, the authors concluded that the model could be a supportive diagnostic tool for evaluating thyroid cartilage invasion in laryngeal and hypopharyngeal cancers [21]. Santin et al. applied a VGG16 machine learning model to a dataset comprising 515 CT images of thyroid cartilage, including 326 images with abnormal thyroid cartilage findings. The model had an AUC of 0.72 in detecting abnormal findings. The authors reported that the model could be useful in identifying abnormal thyroid cartilage features [25]. Hao et al. applied the ResNet-3Dsml model to CT images of 285 patients with laryngeal and hypopharyngeal cancers. The model found an AUC of 0.844 and an accuracy of 80.0% in detecting thyroid cartilage invasion. Given these results, the authors suggested that the model could be used as a cost-effective and non-invasive diagnostic tool for identifying cartilage invasion [26]. In our study, we applied an ensemble model to a dataset of 1251 CT images to detect thyroid cartilage invasion. Consistent with findings in the literature, the model had an accuracy of 96.54% and an AUC value of 0.99. Based on these results, we report that the ensemble model demonstrates a very high level of performance in detecting thyroid cartilage invasion. A summary of radiomic, machine learning studies in detecting neoplastic infiltration of thyroid cartilage is shown in Table 1.

In current practice, MRI and CT imaging are frequently utilized for the detection of cartilage invasion, with their interpretation typically relying on radiologists. However, it is well established that such interpretations can occasionally result in suboptimal or unnecessary treatment decisions. The ensemble model developed in this study effectively reduced the likelihood of both overestimation and underestimation of cartilage invasion by statistically optimizing the outputs of multiple deep learning–based submodels. Analysis of the complexity matrix revealed that the model produced false-positive (overestimation) results in only 8 out of 200 cases and false-negative (underestimation) results in only 5 out of 176 cases. These findings demonstrate that the model can identify the presence of invasion with high sensitivity (97.2%) and specificity (96.0%). Although the ensemble model demonstrated high accuracy in detecting the presence of thyroid cartilage invasion, it currently lacks the capability to distinguish between focal (T3) and extensive (T4) invasion. Therefore, while the model can support radiologists in identifying cartilage involvement more reliably, its contribution to directly preventing suboptimal or unnecessary treatment decisions remains limited. Future studies focusing on stage-specific classification may enhance its clinical applicability in treatment planning. However, the reliability of these models is contingent upon the quality of input data, appropriate feature selection, and rigorous validation using clinically relevant benchmarks. Further multicenter studies with large, heterogeneous cohorts are warranted to substantiate the clinical utility of ensemble-based diagnostic frameworks in this context.

This study has several limitations. First, as reported by Houas et al., thyroid cartilage invasion was observed on computed tomography (CT) in 53.5% of patients with advanced laryngeal squamous cell carcinoma [27]. This finding indicates that a substantial proportion of advanced cases may present without cartilage involvement. Therefore, since the proposed model relies on imaging features associated with cartilage invasion for detection and classification, it may not be applicable to patients without cartilage infiltration. Consequently, the diagnostic performance of the model is limited to cases with potential or existing cartilage invasion.

In addition, as noted by Guo et al., asymmetric ossification of the normal thyroid cartilage can be misinterpreted as thyroid cartilage invasion [24]. Furthermore, the CT attenuation values of nonossified hyaline cartilage are similar to those of tumor tissue, making the assessment of thyroid cartilage invasion on CT challenging. These imaging characteristics may lead to false-positive cartilage invasion findings, both on CT evaluation and within the machine learning model.

In our study, the performance of the deep learning model was compared only with histopathological results, rather than with assessments made by radiologists experienced in head and neck imaging. This represents a significant methodological limitation that reduces the clinical applicability of the model. In addition, this study is limited by its single-institution setting, the lack of heterogeneity in CT imaging protocols, and the relatively small patient cohort. These limitations affect the study’s generalizability. Although CT and MRI remain the standard imaging modalities, their accuracy is constrained by both technical and observer-dependent factors. Given this, Artificial intelligence methods, such as the proposed ensemble model, may enhance diagnostic performance. Furthermore, establishing histopathological correlation with CT images enhances the clinical validity of the results. Finally, the retrospective design and single-center dataset may limit the generalizability of the findings. Future multicenter studies with larger sample sizes and stage-specific classification approaches are warranted to improve the robustness and clinical applicability of the model.

## 5. Conclusions

The ensemble machine learning model based on CT radiomic features demonstrated high accuracy in detecting neoplastic infiltration of the thyroid cartilage. However, it lacks the ability to differentiate between focal (T3) and extensive (T4) involvement. Consequently, while the model can assist radiologists in improving the reliability of cartilage invasion detection, its direct impact on preventing suboptimal or unnecessary treatment decisions remains limited. Future research focusing on stage-specific differentiation may further enhance the model’s clinical utility in treatment planning and decision-making. In conclusion, this model could be integrated into future clinical practice in laryngology and head and neck surgery for the detection of cartilage neoplastic infiltration.

## Figures and Tables

**Table 1:** A summary of radiomic, machine learning studies in detecting neoplastic infiltration of thyroid cartilage.

Author	Method	Data Count (n = Number of Patients, i = Number of Images)	AUC	Accuracy
Guo et al. [24]	LR-SVMSMOTE LR	n = 265	0.905	71.3%
Takano et al. [21]	ResNet101	n = 91	0.82	90%
Santin et al. [25]	VGG16	n = 515	0.72	
Hao et al. [26]	ResNet-3Dsml	n = 285	0.844	80%
Our study	Ensemble (ResNet50, MobileNet)	n = 313	0.99	96.54%

## Data Availability

The datasets used or analyzed during the current study are available from the corresponding author on reasonable request.

## Abbreviations

SVMSMOTE	The support vector machine-based synthetic minority oversampling technique
CT	Computer Tomography
MRI	Magnetic Resonance Imaging
SCC	Squamous Cell Carcinoma
ROC	Receiver Operating Characteristic
AUC	Area Under The Curve
AI	Artificial Intelligence
